# Phase Ia/Ib Study of Afatinib with Capecitabine in Patients with Refractory Solid Tumors and Pancreaticobiliary Cancers

**DOI:** 10.3390/cancers17111830

**Published:** 2025-05-30

**Authors:** Gentry G. King, Kelsey K. Baker, Andrew L. Coveler, William P. Harris, Stacey A. Cohen, Veena Shankaran, David B. Zhen, Rachael A. Safyan, Hannah H. Lee, Annie Alidina, Jeniece Hensel, Reina Hibbert, Greg A. Durm, Yvonne C. LaFary, Anne Younger, Sita Kugel, Eric Collisson, Eric Q. Konnick, Mary W. Redman, Bryan P. Schneider, Colin C. Pritchard, Safi Shahda, Elena Gabriela Chiorean

**Affiliations:** 1 University of Washington School of Medicine, Seattle, WA 98195, USA; gking@fredhutch.org (G.G.K.); acoveler@uw.edu (A.L.C.); wph3@uw.edu (W.P.H.); shiovitz@uw.edu (S.A.C.); vshank@uw.edu (V.S.); dbzhen@uw.edu (D.B.Z.); rsafyan@uw.edu (R.A.S.); hpiek@fredhutch.org (H.H.L.); aalidina@fredhutch.org (A.A.); toroj@fredhutch.org (J.H.); rhibbert@fredhutch.org (R.H.); konnick@uw.edu (E.Q.K.); cpritch@uw.edu (C.C.P.); 2 Fred Hutchinson Cancer Center, Seattle, WA 98109, USA; kkbaker@fredhutch.org (K.K.B.); skugel@fredhutch.org (S.K.); eric@fredhutch.org (E.C.); mredman@fredhutch.org (M.W.R.); 3 Indiana University Melvin and Bren Simon Cancer Center, Indianapolis, IN 46202, USA; gdurm@iu.edu (G.A.D.); ylafary@iu.edu (Y.C.L.); anefoste@iu.edu (A.Y.); bpschnei@iu.edu (B.P.S.); safi.shahda@intelliatex.com (S.S.); 4 Intellia Therapeutics, Cambridge, MA 02139, USA

**Keywords:** afatinib, biliary cancers, capecitabine, EGFR, HER2, *KRAS*, pancreatic cancer

## Abstract

Background: The epidermal growth factor receptor (EGFR) is overactive in many tumors. This phase I trial evaluated the safety and preliminary efficacy of afatinib plus capecitabine in refractory pancreatic ductal adenocarcinoma (PDA), biliary tract cancers (BTC), and other solid tumors. Patients and Methods: The phase Ia study had a 3 + 3 design with capecitabine 1000 mg/m^2^ twice daily on days 1–14 and afatinib 20 mg, 30 mg, or 40 mg daily in 21-day cycles. In phase Ib, 15 patients, each with PDA and BTC, were treated at maximum tolerated dose (MTD). Results: A total of 41 patients were enrolled. No dose-limiting toxicities were observed, and the MTD was 40 mg afatinib plus capecitabine. Among 36 response-evaluable patients, one had a partial response (3%), and eight (22%) had stable disease. Median progression-free survival (PFS) was 1.9 months (95% CI 1.0, 2.0) for PDA and 1.9 months (95% CI 1.6, 3.4) for BTC. Median overall survival (OS) was 3.2 months (95% CI 2.0, 5.8) for PDA, and 4.6 months (95% CI 1.9, 6.1) for BTC. Median OS was 5.8 months (95% CI 2.0, 9.6) for *KRAS*^WT^ PDA, and 5.0 months (95% CI 1.6, 6.1) for *KRAS*^WT^ BTC, vs. 3.9 months (95% CI 1.9, 5.8) for *KRAS*^MUT^ PDA and 3.1 months (95% CI 1.0, 22.8) for *KRAS*^MUT^ BTC, respectively. Conclusions: Afatinib plus capecitabine is tolerable but does not have clinically meaningful efficacy in refractory PDA/BTC. Future studies should test novel anti-EGFR/HER2 therapies in *KRAS*^WT^ cancers further selected with a comprehensive molecular profile.

## Simple Summary

The epidermal growth factor receptor (EGFR) is overactive in many solid tumors. Afatinib, an EGFR/HER2/HER3 inhibitor, synergizes with capecitabine in preclinical models. This phase Ia/Ib trial evaluated the safety and preliminary efficacy of afatinib plus capecitabine in refractory pancreatic ductal adenocarcinoma, biliary cancers, and other solid tumors. Afatinib plus capecitabine is tolerable but does not have clinically meaningful efficacy in refractory pancreatico-biliary cancers. Numerically better disease control rates (50% vs. 20%) and median survival (5.8 months vs. 3.9 months) were observed for *KRAS* wild-type (WT) vs. *KRAS*-mutated (MUT) pancreatic cancers. Similarly, better disease control (40% vs. 20%) and median survival (5 months vs. 3.1 months) were observed for *KRAS*^WT^ vs. *KRAS*^MUT^ biliary cancers. Future studies should test novel anti-EGFR therapies in *KRAS*^WT^ cancers, further selected with a complete molecular profile.

## 1. Introduction

The erythroblastic leukemia viral oncogene homolog (ERBB), also known as the epidermal growth factor (EGF) family of receptors (EGFR), includes EGFR/ERBB1/HER1, ERBB2/HER2, ERBB3/HER3, and ERBB4/HER4. Overexpression of the ERBB/EGFR family of proteins occurs in up to 90% of pancreatic ductal adenocarcinomas (PDA) and 60% of biliary tract cancers (BTC) and has been associated with more aggressive biology and inferior survival [1,2]. PDA stands as one of the most aggressive cancers, with median overall survival (OS) rates of 11, 6, and 3 months, respectively, for patients with metastatic disease treated with first-, second- and third-line therapies [3,4,5]. Biliary tract cancers are a heterogeneous group of malignancies including cholangiocarcinomas and gallbladder cancers, with broader therapeutic options, but still have a poor prognosis, with a median OS of 13 months with first-line chemo-immunotherapy, and 6 and 4 months, respectively, with second- and third-line regimens for metastatic disease [6,7,8]. Precision oncology has reshaped treatment paradigms across multiple cancers, including pancreaticobiliary cancers with actionable alterations, offering new hope through biomarker-driven therapies [5,7].

The first-generation anti-EGFR small-molecule tyrosine kinase inhibitor (TKI) erlotinib demonstrated minimal but statistically significant improved overall survival (OS) in combination with gemcitabine vs. gemcitabine alone as a first-line treatment of patients with advanced PDA (6.2 vs. 5.9 months, HR = 0.82, *p* = 0.03) [9]. However, a retrospective analysis of 117 patients with archival tumors available for *KRAS* mutation testing noted superior OS with erlotinib in *KRAS* wild-type (*KRAS*^WT^) vs. *KRAS*-mutated (*KRAS*^MUT^) PDA (6.1 vs. 4.5 months, HR = 0.66, *p* = 0.34) [10]. Similarly, the German AIO-PK0104 trial, which evaluated gemcitabine or capecitabine plus erlotinib as a first-line treatment of advanced PDA, found that *KRAS*^WT^ PDA had higher OS than *KRAS*^MUT^ cancers (7.9 vs. 5.7 months, HR = 1.68, *p* = 0.005) [11]. More recently, the randomized study with the anti-EGFR antibody nimotuzumab plus gemcitabine vs. gemcitabine alone in *KRAS*^WT^ PDA (n = 90) showed OS benefit (10.9 vs. 8.5 months, HR = 0.62; *p* = 0.04) [12]. Several reports also indicate benefit from anti-EGFR TKIs in patients with *KRAS*^WT^ PDA with concurrent *EGFR* activating mutations [13,14]. A similar challenge exists for BTC, where large randomized trials have not shown a significant survival advantage of erlotinib in combination with chemotherapy vs. chemotherapy alone, despite higher response rates and a higher proportion of patients with *KRAS*^WT^ tumors (70–80%) [15,16].

In preclinical models, 5′-deoxy-5-fluorouridine (5′-DFUR) in combination with gefitinib or lapatinib has synergistic activity thought to be due to EGFR TKI-mediated upregulation of thymidylate phosphorylase, the enzyme that converts 5′-DFUR into the active metabolite 5-fluorouracil (5-FU) [17,18,19]. Afatinib is a selective, highly potent second-generation EGFR TKI, which irreversibly blocks the ERBB family of proteins and has superior efficacy to erlotinib in preclinical models of PDA [20]. It has also been shown to enhance fluoropyrimidine antitumor activity by downregulating thymidine synthase [21]. Afatinib has several advantages over first-generation EGFR TKIs, including broader inhibition of the EGFR, HER2, and HER4 kinases, irreversible and more sustained kinase inhibition, and activity in cells resistant to first-line EGFR inhibitors [22]. Given preclinical synergism and clinical interest in testing more potent EGF pathway signaling inhibitors, we wanted to assess the safety and efficacy of the combination of afatinib with capecitabine in patients with refractory solid tumors and pancreaticobiliary cancers and perform next-generation genomic sequencing to identify molecular markers, including *ERBB* aberrations, predictive of benefits.

## 2. Materials and Methods

### 2.1. Patient Selection

Eligible patients were ≥18 years, with histologically proven advanced solid malignancies (phase Ia) or PDA and BTC (phase Ib), an Eastern Cooperative Oncology Group (ECOG) performance status of 0 to 2 (phase Ia) or 0 to 1 (phase Ib), any number of prior therapies (phase Ia) or up to two prior therapies (phase Ib), life expectancy ≥ 12 weeks, and adequate organ function. Prior treatment with erlotinib, gefitinib, or EGFR-blocking monoclonal antibodies (cetuximab and panitumumab) was permitted. There was no requirement for patients to have tumors with known *EGFR/HER2/HER3* gene amplification, mutations, or *KRAS*^WT^ status to be eligible for enrollment. Exclusion criteria included prior treatment with afatinib, brain metastases, unstable cardiovascular and cerebrovascular events within 3 months of registration, active venous thrombosis, anti-cancer systemic therapy within 21 days of registration, major surgery or non-palliative radiation within 30 of registration, and impairment of gastrointestinal function, which could affect administration and absorption of oral drugs. All patients provided written informed consent approved by the University of Washington School of Medicine and by the Indiana University School of Medicine Institutional Review Boards, and this study was conducted in accordance with the International Conference on Harmonization Good Clinical Practice guidelines.

### 2.2. Study Design and Treatment

This was an open-label, non-randomized phase Ia/Ib study whereby the phase Ia portion enrolled patients with solid tumors in a “3 + 3” design, and the phase Ib portion enrolled 30 patients with pancreaticobiliary cancers (15 each BTC and PDA) at the MTD. Capecitabine was administered orally at 1000 mg/m^2^ twice daily (BID) on days 1–14 and afatinib was dosed orally daily (QD) in escalating cohorts: 20 mg (cohort 1), 30 mg (cohort 2), and 40 mg (cohort 3) in 21-day cycles in phase Ia, and at MTD in phase Ib.

Safety assessments, including a physical exam and laboratory assessments, were conducted on day 1 of every 21-day cycle and on day 14 of cycle 1. Adverse events (AEs) were graded using Common Terminology Criteria for Adverse Events (CTCAE), version 3.0. Tumor response was assessed every 9 weeks according to RECIST version 1.1.

Molecular profiling for genomic alterations with next-generation sequencing (NGS) was performed on archival tumor tissue or fresh core biopsies if archival tumor samples were not available; liquid NGS was allowed if tumor NGS could not be performed. If patients had already undergone molecular tumor profiling using institutional or commercial next-generation sequencing assays, NGS reports were collected.

### 2.3. Statistical Analysis

In phase Ia, patients were enrolled and treated in three dose levels, with a “3 + 3” design. In phase Ib, 15 patients with pancreatic adenocarcinoma and 15 patients with biliary tract cancers were enrolled and treated at MTD.

In phase Ia, dose escalation was allowed if dose-limiting toxicity (DLT) occurred in 0/3 or ≤1/6 DLT evaluable patients in each cohort during cycle 1. DLTs were evaluated during cycle 1 and were defined as follows: grade 4 thrombocytopenia persisting more than 4 days or with bleeding requiring platelet transfusion, grade 4 neutropenia lasting more than 7 days, grade 3 or 4 febrile neutropenia (≥38.5 °C), treatment-related toxicity that resulted in more than 7 days of missed afatinib doses, and any grade 3 or 4 non-hematologic toxicity related to the combination (occurring despite optimal supportive care, if applicable) except grade 3 deep venous thrombosis and alopecia. Patients were considered DLT evaluable if they completed at least one cycle of treatment. Treatment continued until disease progression, unacceptable toxicity, or withdrawal of consent.

Antitumor activity was evaluated in all patients enrolled in phase Ia and phase Ib portions of the study by overall response rate (ORR = complete response [CR] plus partial response [PR]), stable disease (SD), duration of response (DoR), and disease control rate (DCR = ORR + SD), which were assessed in patients who had at least one imaging scan post-treatment initiation or had documented clinical disease progression in the absence of a scan. Progression-free (PFS) and overall survival (OS) were assessed in all enrolled patients (intention-to-treat population) as well as in response-evaluable patients. Response-evaluable patients will be considered those who complete the first response evaluation after 3 cycles of therapy, as well as patients who discontinue treatment prior to cycle 3 due to disease progression. Responses were tabulated. PFS and OS estimates were calculated using the Kaplan–Meier method. PFS was defined as the time from enrollment to the earliest of progression or death. OS was defined as the time from enrollment to death. Patients last known to be alive (OS, PFS) and progression free (OS) were censored at the date of last contact.

A descriptive analysis of the patients’ tumor molecular profile and their relationship with outcomes (OS, PFS, best response) was conducted.

## 3. Results

### 3.1. Patient Characteristics

A total of 41 eligible patients were enrolled at two academic centers: the University of Washington/Fred Hutchinson Cancer Center and Indiana University Melvin and Bren Simon Cancer Center, from November 2016 to October 2021. Patients’ baseline characteristics are summarized in Table 1. Eleven patients were enrolled in the phase Ia dose-escalation stage, and 30 patients were enrolled in the phase Ib dose-expansion stage. Patients were treated with a median of 3 cycles (range 1–12) of therapy in phase Ia, and 2 cycles (range 1–8) of therapy in phase Ib.

### 3.2. Dose Escalation and Toxicities

In phase Ia, 9/11 patients were evaluable for DLTs, and all patients were evaluable for safety. Two patients were not evaluable for DLTs due to 2 weeks of missed afatinib doses in cycle 1 unrelated to toxicity (n = 1, cohort 1) and due to death from disease progression (n = 1, cohort 3). Among 9 patients evaluable for DLTs, no DLTs were observed. Thus, the MTD was afatinib 40 mg PO QD with capecitabine 1000 mg/m^2^ PO BID days 1–14 every 21 days. In phase Ib, all 30 patients were evaluable for safety.

The most common treatment-emergent adverse events (TEAE) were diarrhea (68%), nausea (63%), and oral mucositis (61%). TEAEs occurring in more than 15% of patients are listed in Table S1. The most common treatment-related adverse events (TRAEs) were diarrhea (68%), oral mucositis (58%), nausea (54%), and fatigue (51%) (Table 2). Nine patients (22%) experienced grade 3 TRAE, of which the most common were diarrhea (12%) and nausea (5%), and no grade 4 or 5 TRAEs were observed.

Reasons for study treatment discontinuation in phase Ia were disease progression (11/11, 100%), and in phase Ib, disease progression (22/30, 73%), AEs (3/30, 10%), other causes unrelated to treatment (3/30, 10%), and withdrawal of consent (2/30, 7%).

### 3.3. Antitumor Activity and Survival Outcomes

**Figure 1:**
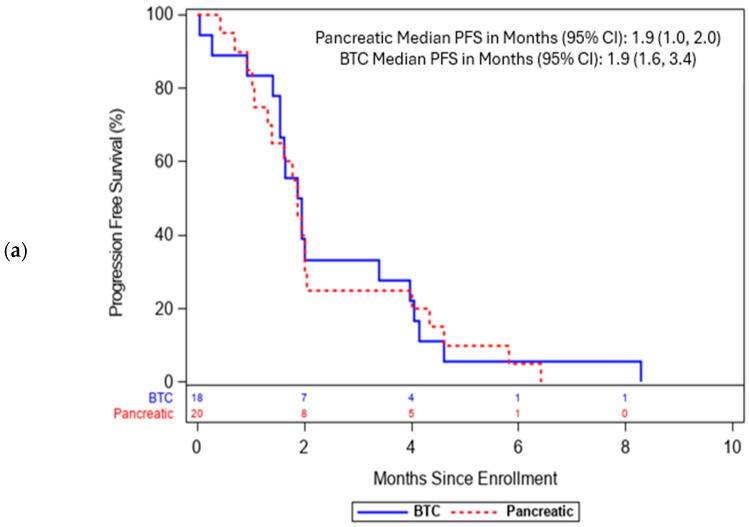

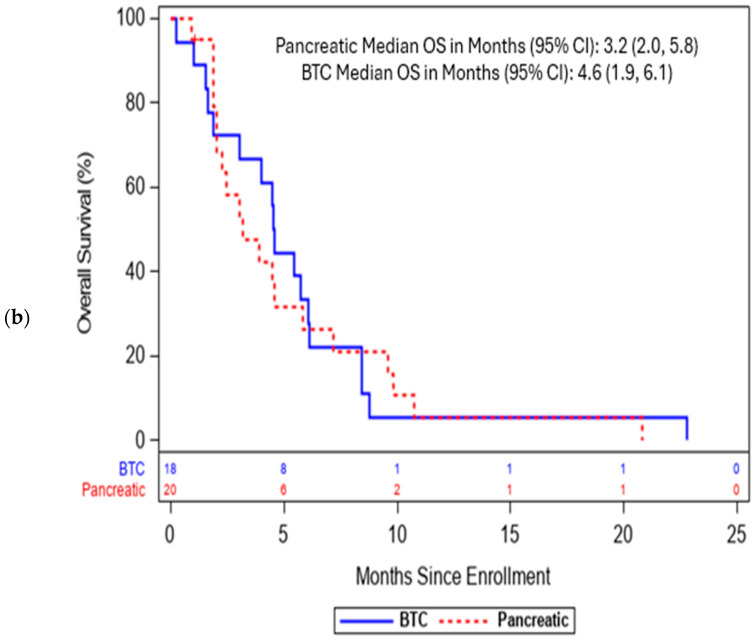
Progression-free and overall survival for pancreatic cancer (PDA) and biliary tract cancer (BTC) patients from time of study enrollment (**a**) Kaplan–Meier estimates for progression-free survival (PFS) for pancreatic cancer and BTC Patients (**b**) Kaplan–Meier estimates for overall survival (OS) among pancreatic cancer and BTC patients.

Of the 41 patients enrolled, 36 were evaluable for response (Table 3). Five patients were not response evaluable due to early discontinuation due to toxicity (n = 3) or withdrawal of consent (n = 2). The objective response rate (ORR) was 3% (1/36) with one partial response (unconfirmed) in a BTC patient with *KRAS*^WT^, *EGFR*-amplified cholangiocarcinoma. Eight patients (22%, four patients each, with BTC and PDA) had SD; thus, the DCR was 25% (9/36). Secondary endpoints of PFS and OS were analyzed for patients enrolled in the phase Ia and Ib with PDA (n = 20) and BTC (n = 18). The median PFS for patients with PDA (n = 20) was 1.9 months (95% CI 1.0, 2.0), and for patients with BTC (n = 18) it was 1.9 months (95% CI 1.6, 3.4) (Figure 1a). The median OS for PDA patients was 3.2 months (95% CI 2.0, 5.8) and for BTC patients it was 4.6 months (95% CI 1.9, 6.1) (Figure 1b).

### 3.4. Genomic Analysis and Correlative Studies

Of the 41 patients enrolled, 39 patients had sufficient tumor quantity (n = 38) or had blood testing (n = 1) for NGS profiling (n = 19 PDA, n = 17 BTC, and n = 1 each fibrolamellar hepatocellular carcinoma, gastric, and esophageal adenocarcinomas). Molecular profiling was conducted with FoundationOne (Foundation Medicine, Cambridge, MA, USA, n = 22), UW-OncoPlex^TM^ (University of Washington, Department of Laboratory Medicine, Seattle, WA, USA, n = 8), GPS Cancer^TM^ (NantOmics, Culver City, CA, USA, n = 5), Tempus xT (Chicago, IL, USA, n = 2), StrataNGS^TM^ (Strata Oncology, Ann Arbor, MI, USA, n = 1), and CellNetix NGS (CellNetix Pathology & Laboratories, Seattle, WA, USA, n = 1) NGS assays.

**Figure 2:**
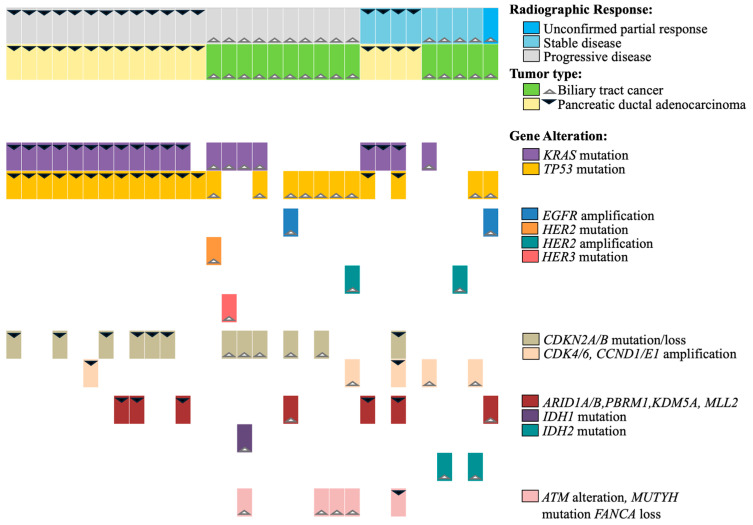
Map of genomic alterations and best response for patients with pancreatic and biliary tract cancers.

*KRAS* mutations were present in 17/19 (89.5%) PDA and in 5/17 (29.4%) BTC. Among the NGS-evaluable population, alterations in *TP53* (73.7%) and cell cycle genes *CDKN2A/B* (36.8%) and *CCNE1* (13.2%) were most common, but defects in chromatin remodeling complex genes (e.g., *ARID1A/B, PBRM1, KDM5A, KMT2D/MLL2*) (18.4%) and the PI3K/Akt/mTOR pathway (10.2%) were also frequently observed. Genomic alterations in the EGFR/HER2/HER3 pathway were present in 8/39 (20%) tumors: seven BTCs (2 *EGFR* amplification, three *HER2* amplification, one *HER2* mutation, one *HER3* mutation), one esophageal (*HER2* mutation), and one PDA had HER3 protein amplification by proteomic testing without *HER3* gene amplification. Of these, seven tumors were *KRAS*^WT^ and two BTCs had co-occurring *HER3* and *KRAS* G13D, or *HER2* and *KRAS* K117N mutations. No cancers had *EGFR* activating mutations. A summary of tumor genomic alterations and best response for PDA/BTC patients is provided in Figure 2 and for all patients in Table S2.

**Figure 3:**
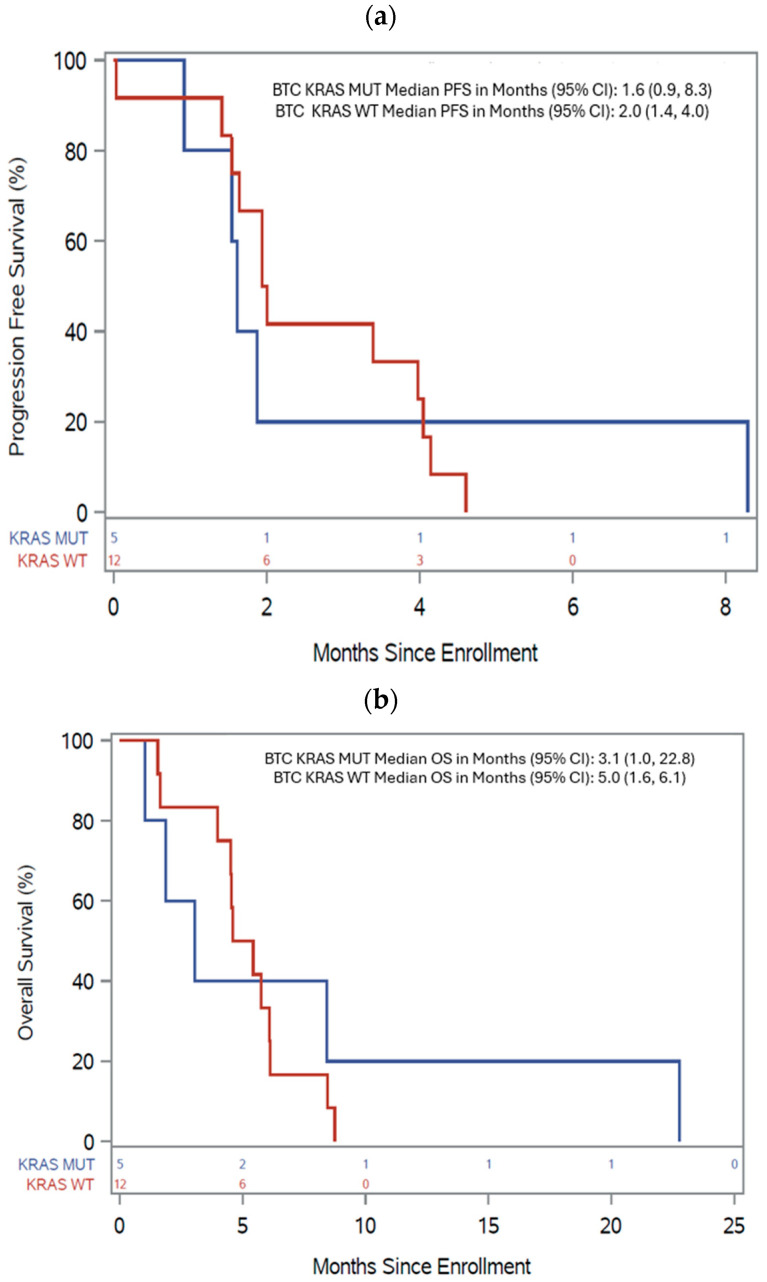

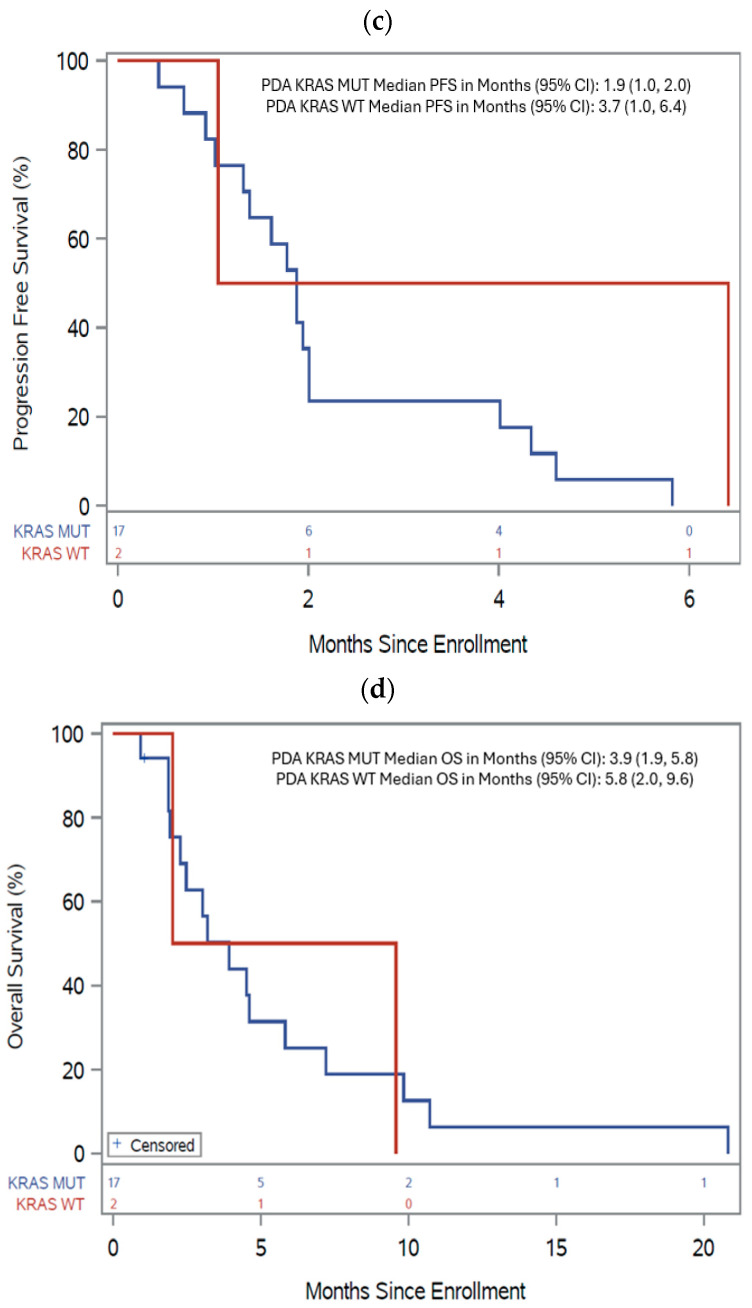
Progression-free and overall survival for *KRAS* wild-type (*KRAS*^WT^, red lines) and *KRAS*-mutated (*KRAS*^MUT^, blue lines) biliary tract cancer (BTC) and pancreatic cancer (PDA) patients. (**a**) Kaplan–Meier estimates for progression-free survival (PFS) for patients with *KRAS*^WT^ and *KRAS*^MUT^ BTC. (**b**) Kaplan–Meier estimates for overall survival (OS) for patients with *KRAS*^WT^ and *KRAS*^MUT^ BTC. (**c**) Kaplan–Meier estimates for PFS for patients with *KRAS*^WT^ and *KRAS*^MUT^ PDA. (**d**) Kaplan–Meier estimates for OS for patients with *KRAS*^WT^ and *KRAS*^MUT^ PDA.

Seventeen BTC patients had tumors with NGS molecular analysis: 12 *KRAS*^WT^ and 5 *KRAS*^MUT^. All patients were evaluable for survival, and 15 were evaluable for response. Among ten response-evaluable *KRAS*^WT^ BTC patients, the best response was one uPR (10%; *EGFR* amplified), three SD (30%; one *HER2* amplified, two with no *EGFR/HER2/HER3* alterations), and the DCR was 40%. Among five response-evaluable *KRAS*^MUT^ BTC patients, the best response was one SD (20%, *FGFR1* amplified), and the DCR was 20%. The median PFS for patients with *KRAS*^WT^ vs. *KRAS*^MUT^ BTC was 2.0 months (95% CI 1.4, 4.0) vs. 1.6 months (95% CI 0.9, 8.3) (Figure 3a), and the median OS was 5.0 months (95% CI 1.6, 6.1) vs. 3.1 months (95% CI 1.0, 22.8) (Figure 3b), respectively. *KRAS*^WT^ BTC with or without *EGFR/HER2* amplification had a DCR of 50% (2/4) vs. 30% (2/6), median PFS of 4.0 months (95% CI 1.6, 4.6) vs. 1.9 months (95% CI 0.0, 3.4), and median OS of 4.6 months (95% CI 1.6, 6.1) vs. 5.4 months (95% CI 1.6, 8.4).

Nineteen PDA patients had tumors with NGS molecular analysis available, of which two were *KRAS*^WT^ and 17 *KRAS*^MUT^. All patients were evaluable for survival and 17 were evaluable for response. Among *KRAS*^WT^ PDA patients, one had SD (50%, no *EGFR/HER2/HER3* genomic alterations but positive HER3 protein overexpression by GPS Cancer^TM^ proteomic analysis, (NantOmics, Culver City, CA, USA)) and one with *HER2*-amplified PDA had progressive disease (PD). Among 15 response-evaluable *KRAS*^MUT^ PDA patients, three patients had SD (20%). The median PFS for patients with *KRAS*^WT^ vs. *KRAS*^MUT^ PDA was 3.7 months (95% CI 1.0, 6.4) vs. 1.9 months (95% CI 1.0, 2.0) (Figure 3c), and the median OS was 5.8 months (95% CI 2.0, 9.6) vs. 3.9 months (95% CI 1.9, 5.8) (Figure 3d), respectively.

## 4. Discussion

This phase Ia/Ib study demonstrated the safety and tolerability of afatinib in combination with capecitabine in advanced pancreaticobiliary cancers and other solid tumors. The MTD was afatinib 40 mg PO QD in combination with capecitabine 1000 mg/m^2^ PO BID days 1–14 given in 21-day cycles. Toxicities were consistent with those expected from afatinib and capecitabine: diarrhea and mucocutaneous AEs, including palmar–plantar erythrodysesthesia, were predominant, but only three patients (7%) stopped study treatment due to related toxicities. Despite adequate tolerability of this regimen among patients with advanced pancreaticobiliary cancers, objective anti-tumor activity was limited. Median PFS of 1.9 months and median OS of 3.2 and 4.6 months, respectively, for PDA and BTC in the third-line treatment setting were comparable with historical data for refractory PDA/BTC [23,24]. Therefore, despite preclinical synergism, we observed no evidence of meaningful clinical activity from afatinib plus capecitabine.

Biomarker selection for patients most likely to benefit from EGFR/HER2 signaling inhibition continues to be worth exploring in studies with EGFR/HER2 inhibitor combinations. For instance, systemic treatment of pancreaticobiliary cancers has diversified and improved significantly over recent years, in part due to biomarker identification, with several therapies having been FDA approved based on targetable molecular alterations. *HER2* amplification, but mostly HER2 protein overexpression by IHC, has been shown by several key studies (MyPathway, HERIZON-BTC-01, SGNTUC-019, DESTINY-PanTumor02) to be a valid therapeutic target for anti-HER2 monoclonal antibodies, TKIs, and antibody-drug conjugates, with ORRs averaging 40% in a tumor-agnostic population including BTC [25,26,27,28]. In contrast, HER2-targeted therapy in *HER2*-amplified PDA has only limited activity, which may be due, at least in part, to a higher frequency of *KRAS* mutations in this disease [28,29,30].

Our study enrolled patients unselected for molecular alterations, but 39 patients in all, and 36 patients with pancreaticobiliary cancers had available genomic analysis by NGS. Only one patient with BTC (*EGFR* amplified, *KRAS*^WT^ cholangiocarcinoma) achieved an unconfirmed PR, and eight patients (three *KRAS*^WT^ BTC, of whom one was *HER2* amplified, one *KRAS*^WT^ PDA with HER3 protein overexpression but not *HER3* amplified by NGS, three *KRAS*^MUT^ PDA, and one *KRAS*^MUT^ BTC) had SD. We tested *HER2* amplification via NGS obtained from archival tumor samples, but patients did not undergo baseline tumor or liquid biopsies to assess HER2 status before study treatment. Whereas the degree of concordance between *HER2* amplification by NGS and HER2 protein overexpression by IHC is generally high (>90%) [31], it varies by tumor type [32], and approximately 30% discordance occurs between baseline and subsequent tumor testing [32]. Thus, the lack of study baseline HER2 testing could explain why several BTC/PDA patients with *HER2*-amplified tumors by archival testing did not benefit from afatinib-based therapy. While one patient with *EGFR*-amplified BTC had PR, another patient with *EGFR*-amplified BTC had PD; thus, we cannot infer that *EGFR* amplification is a strong correlative biomarker for response to afatinib. Moreover, molecular profile analysis of patients from the randomized phase III AIO-PK0104 study of gemcitabine with or without erlotinib in pancreatic cancer did not demonstrate a correlation between *EGFR* amplification (18%) or EGFR overexpression (46%) with benefit from erlotinib [11]. The CTG PA.3 similarly did not show any association between *EGFR* amplification and survival benefit, despite showing amplification or high-polysomy in 47% of pancreatic cancer patients [10]. In BTC, the incidence of *EGFR* amplification is approximately 6%, and prospective clinical data to show predictive benefit to EGFR-targeted treatment is also limited [33].

*KRAS* mutations, encountered in approximately 20–30% BTC and 90% PDA [34,35,36], confer resistance to anti-EGFR targeting therapies due to downstream constitutive activation of *RAS* signaling. While *KRAS/NRAS* mutations are well known to predict a lack of efficacy from anti-EGFR antibodies in colorectal cancers [37,38], studies investigating the addition of the EGFR TKI erlotinib to chemotherapy in advanced BTC and PDA suggested a similar pattern [10,11,16]. As expected, most *KRAS*^WT^ tumors in our study were BTC (n = 12) and only two were PDA.

Although our study is limited by small patient numbers, we observed numerically higher DCR of 50% vs. 20% and median OS of 5.8 vs. 3.9 months in *KRAS*^WT^ vs. *KRAS*^MUT^ pancreatic cancers, and higher DCR of 40% vs. 20% and median OS of 5.0 vs. 3.1 months in *KRAS*^WT^ vs. *KRAS*^MUT^ biliary tract cancers, respectively. In addition, higher median PFS (4.0 vs. 1.9 months) but not OS (4.6 vs. 5.4 months) occurred among patients with *KRAS^WT^ EGFR/HER2* amplified vs. *EGFR/HER2* non-amplified BTC.

Nevertheless, we also observed clinical benefit in some *KRAS*^MUT^ tumors. For example, a patient with *KRAS*^MUT^ *FGFR1*-amplified BTC had durable stable disease, PFS of 8 months, and OS of 22.8 months, despite the known association of activated FGFR1 signaling with resistance to EGFR-targeted therapies [39]. Three patients with *KRAS*^MUT^ PDA achieved SD, of whom one had a tumor with *ATM* defects, and one had SWI/SNF complex alterations in *PBRM1* and *ARID1B* genes. These genomic aberrations have all been associated with intrinsic or acquired resistance, leading to reduced benefit from EGFR/HER2 inhibition [40,41,42]. While preclinical efficacy from afatinib in pancreatic cancer has been suggested to correlate with EGFR/HER2/HER3 expression and occurred irrespective of *KRAS* status [43], disease stabilizations seen in *KRAS*^MUT^ tumors in our study may have been solely due to capecitabine chemotherapy.

Although it could be appealing to further investigate the combination of afatinib and capecitabine in *KRAS*^WT^ BTC patients based on these results, patient numbers were low, and previous larger randomized phase II studies (PICCA, Vecti-BIL, Taiwan Cooperative Oncology Group-TCOG did not demonstrate improvement in survival or responses with the addition of anti-EGFR monoclonal antibodies panitumumab or cetuximab to chemotherapy vs. chemotherapy alone in *KRAS^WT^* BTC [44,45,46], albeit the phase III NOTABLE study with nimotuzumab added to gemcitabine in *KRAS^WT^* PDA was positive [12]. These data suggest that *KRAS*^WT^ status alone is not sufficient for antitumor efficacy, and other molecular pathways may sustain cancer growth and proliferation. A retrospective biomarker analysis of patients enrolled in the TCOG study treated with gemcitabine and oxaliplatin (GEMOX) with or without cetuximab identified aberrant expression of *ROS1*, *ALK,* or *c-MET* to be associated with poor response to treatment and poor survival [47]. Whereas no patients in our study had tumors harboring these genetic defects, some of which are better captured by RNA sequencing, it is likely that negative selection for *ROS1/ALK/MET* defects may increase the likelihood of benefit from anti-EGFR/HER2 therapy. Similarly, negative hyper-selection for mutations in the *KRAS*, *NRAS, PTEN,* and *EGFR* extracellular domain; amplifications of *HER2* and *MET*; and fusions in *ALK*, *RET,* and *NTRK1* captured by circulating tumor DNA (ctDNA) helped identify colorectal cancer patients with increased benefit from the anti-EGFR antibody panitumumab in the phase III PARADIGM study [48].

In contrast to BTC, where most cancers are *KRAS^WT^*, *KRAS^WT^* PDA comprises only 5–10% of all PDA [36]. However, emerging data describe *KRAS^WT^* PDA as biologically and molecularly distinct from *KRAS^MUT^* PDA, showing a higher prevalence of microsatellite instability-high, tumor mutation burden-high, gene fusions, DNA damage repair defects, and other targetable oncogenic alterations [49]. In fact, an integrative analysis of metastatic PDA in the PanGen trial revealed that the mutation and expression-based profile of *KRAS^WT^* PDA is more similar to cholangiocarcinoma compared to *KRAS^MUT^* PDA [50]. Alterations in the EGFR/HER2 signaling pathway are more likely to be encountered in *KRAS*^WT^ PDA (6.8%) vs. *KRAS*^MUT^ PDA (1.7%) [51]. Interestingly, although there were only two *KRAS*^WT^ PDA in our study, one had HER3 protein overexpression, and none of the *KRAS*^MUT^ PDA had *EGFR/HER2/HER3* alterations.

In addition to significant benefit in treatment-naïve non-small cell lung cancer (NSCLC) with non-resistant EGFR mutations, afatinib has shown notable efficacy in tumors, including PDA, harboring neuregulin (*NRG1*) fusions, as seen in the basket study TAPUR [52]. Afatinib has been previously tested in several clinical trials for patients with pancreaticobiliary cancers, with no survival benefit, but none of the studies provided correlative molecular testing (Table 4). Afatinib plus gemcitabine was tested against gemcitabine alone in the randomized phase 2 Arbeitsgemein schaft Internistische Onkologie (AIO) ACCEPT study as first-line treatment for metastatic PDA, with no OS or PFS benefit [53]. The AIO group subsequently tested afatinib with gemcitabine plus nab-paclitaxel as first-line therapy for metastatic PDA in the phase 1b AFFECT study, with no benefit compared to historical data [54]. Afatinib was also combined with gemcitabine plus cisplatin as first-line treatment for BTC, but OS and PFS rates were not superior to chemotherapy historical controls [55]. Whereas these studies did not include comprehensive molecular NGS testing, afatinib did not seem to add any significant efficacy vs. standard chemotherapy in biomarker-unselected pancreaticobiliary tumors. Nevertheless, in tumors with ERBB/HER family mutations or amplifications, afatinib provided modest efficacy, mostly seen in salivary gland cancers, suggesting tumor-dependent activity [56].

With contemporary NGS profiling employing both DNA and RNA sequencing, and selective IHC testing becoming standard-of-care for advanced pancreaticobiliary cancers, precision oncology has the potential to tailor molecular alterations observed in tumor or liquid biopsy samples to appropriate targeted therapies, many of which can induce durable responses and disease stabilizations. Furthermore, new-generation EGFR multi-targeted therapies, including bispecific antibodies, may have enhanced efficacy compared to older EGFR TKIs. Rational therapeutic approaches must be based on understanding the oncogenic drivers and resistance mechanisms underlying each tumor’s biology.

## 5. Conclusions

In conclusion, the combination of afatinib and capecitabine demonstrated acceptable tolerability, with expected diarrhea, nausea, and cutaneous toxicities, but efficacy was limited in refractory solid tumors. While slightly higher disease control and median OS rates were observed in *KRAS*^WT^ pancreaticobiliary tumors, obtaining meaningful clinical benefit from EGFR/HER2 pathway inhibitors in pancreatic and biliary cancers will require further selection with a more comprehensive, novel, and functionally relevant molecular biomarker profile.

## Figures and Tables

**Table 1:** Baseline patient characteristics.

	Phase Ia	Phase Ib
Characteristic, n (%)	n = 11	n = 30
Age at Enrollment, median (range)	63 (24–78)	65 (31–80)
Sex		
Female	3 (27.3)	17 (56.7)
Male	8 (72.7)	13 (43.3)
ECOG performance status		
0	3 (27.3)	9 (30)
1	8 (72.7)	21 (70)
Race		
Asian	1 (9)	2 (6.6)
African American	1 (9)	1 (3.3)
Pacific Islander/Other	0 (0)	1 (3.3)
Caucasian	2 (18.2)	3 (10)
White	7 (63.6)	23 (76.7)
Primary Cancer		
Biliary Tract	3 (27.3)	15 (50)
Fibrolamellar HCC	1 (9)	0 (0)
Gastroesophageal Junction	1 (9)	0 (0)
Gastric	1 (9)	0 (0)
Pancreatic	5 (45.5)	15 (50)
TNM stage		
Stage III	1 (9)	3 (10)
Stage IV	10 (90.9)	27 (90)
Prior Lines of Systemic Therapy		
1	2 (18.2)	8 (26.7)
2	4 (36.4)	19 (63.3)
3+	5 (45.5)	3 (10)

**Table 2:** Maximum-grade treatment-related adverse events that occurred in ≥10% of the patients.

	Phase Ia, n = 11		Phase Ib, n = 30		Total n = 41
Related AEs, n (%)	All	Grade ≥ 3	All	Grade ≥ 3	All
Diarrhea	6 (54%)	0 (0%)	22 (73%)	5 (17%)	28 (68%)
Oral Mucositis	6 (54%)	0 (0%)	18 (60%)	1 (3%)	24 (58%)
Nausea	3 (27%)	0 (0%)	19 (63%)	2 (7%)	22 (54%)
Fatigue	5 (45%)	0 (0%)	16 (53%)	1 (3%)	21 (51%)
Rash	4 (36%)	0 (0%)	16 (53%)	0 (0%)	20 (41%)
Anorexia	2 (18%)	0 (0%)	15 (50%)	1 (3%)	17 (41%)
Vomiting	3 (27%)	0 (0%)	13 (43%)	0 (0%)	16 (39%)
Palmar–plantar erythrodysesthesia (PPE)	6 (54%)	0 (0%)	7 (23%)	1 (3%)	13 (32%)
Dysgeusia	3 (27%)	0 (0%)	7 (23%)	0 (0%)	10 (24%)
Epistaxis	0 (0%)	0 (0%)	7 (23%)	0 (0%)	7 (17%)
Abdominal Pain	4 (36%)	0 (0%)	3 (10%)	0 (0%)	7 (17%)
Peripheral Neuropathy	1 (9%)	0 (0%)	3 (10%)	0 (0%)	4 (10%)
Abnormal Liver Enzymes	0 (0%)	0 (0%)	3 (10%)	0 (0%)	3 (7%)
Back Pain	0 (0%)	0 (0%)	3 (10%)	0 (0%)	3 (7%)
Muscle Weakness	0 (0%)	0 (0%)	3 (10%)	0 (0%)	3 (7%)
Regurgitation	0 (0%)	0 (0%)	3 (10%)	0 (0%)	3 (7%)
Flatulence	2 (18%)	0 (0%)	0 (0%)	0 (0%)	2 (5%)

**Table 3:** Best response in response-evaluable patients.

	Phase Ia	Phase Ib		All Patients
	n = 11	PDA n = 12	BTC n = 13	N = 36
Best Response				
Complete Response	0 (0%)	0 (0%)	0 (0%)	0 (0%)
Partial Response	0 (0%)	0 (0%)	1 (8%)	1 (3%)
Stable Disease	2 (18%)	3 (25%)	3 (23%)	8 (22%)
Progressive Disease	9 (82%)	9 (75%)	9 (69%)	27 (75%)
Objective Response Rate [CR, PR]	0/11 (0%)	0/12 (0%)	1/13 (8%)	1 (3%)
Disease Control Rate [CR, PR, SD]	2/11 (18%)	3/12 (25%)	4/13 (31%)	9 (25%)

**Table 4:** Summary of studies with Afatinib in pancreatic cancer and biliary tract cancers.

Tumor Type	Study Phase	Treatment	Line of Treatment	OS/PFS
PDA [53]	2	Afatinib + Gemcitabine vs. Gemcitabine	First line	OS 7.3 vs. 7.4 mo PFS 3.9 vs. 3.9 mo
PDA [54]	1	Afatinib + Gemcitabine + nab-Paclitaxel	First line	OS 7.5 mo PFS 3.5 mo
BTC [55]	1	Afatinib + Gemcitabine + Cisplatin	First line	OS 7.7 mo PFS 6 mo
PDA * KRAS^WT^ PDA	1	Afatinib + capecitabine	Third line	OS 3.2 mo PFS 1.9 mo
				OS 5.8 mo PFS 3.7 mo
BTC * KRAS^WT^ BTC	1	Afatinib + capecitabine	Third line	OS 4.6 mo PFS 1.9 mo
				OS 5.0 mo PFS 2.0 mo
*NRG1*^+^ PDA [52]		Afatinib	Fourth line	OS 22 mo PFS 15 mo

Abbreviations: * current study; BTC, biliary tract cancers; *KRAS*^WT^, *KRAS* wild type; *NRG1*^+^ *NRG1* fusion positive; OS, overall survival; PDA, pancreatic ductal adenocarcinoma; PFS, progression-free survival.

## Data Availability

Trial data are collected, managed, stored, shared, and archived according to the Fred Hutchinson Cancer Center standard operating procedures to ensure the enduring quality, integrity, and use of the data. Formal requests for data sharing are considered with due regard given to funder and sponsor guidelines. Data will be available after this study’s publication. Requests are via a standard pro forma describing the nature of the proposed research and the extent of data requirements. Data recipients are required to enter a formal data sharing agreement that describes the conditions for release and requirements for data transfer, storage, archiving, publication, and intellectual property.

## Supplementary Materials

The following supporting information can be downloaded at https://www.mdpi.com/article/10.3390/cancers17111830/s1. Table S1: Treatment Emergent Adverse Events Which Occurred in ≥15% of the Patients by Study Phase; Table S2: Genomic Profile and Best Response.
