# Supplementary material for: Phase Ia/Ib Study of Afatinib with Capecitabine in Patients with Refractory Solid Tumors and Pancreaticobiliary Cancers
Source: Cancers (Basel). 2025 May 30;17(11):1830. doi: 10.3390/cancers17111830 (PMC12153597; doi:10.3390/cancers17111830)
Supplement: Supplementary file 1 [file cancers-17-01830-s001.zip › cancers-3560723-supplementary.pdf]

cancers

MDPI

Supplemental Table S1. Treatment Emergent Adverse Events Which Occurred in ≥15% of the Patients by Study Phase.

	Phase Ia, n=11		Phase Ib, n=30		Total
**AEs, n (%)**	**All**	**Grade ≥ 3**	**All**	**Grade ≥ 3**	**n=41**
Diarrhea	6 (54%)	0 (0%)	22 (73%)	5 (17%)	28 (68%)
Nausea	4 (36%)	0 (0%)	22 (73%)	2 (7%)	26 (63%)
Oral mucositis	7 (64%)	0 (0%)	18 (60%)	1 (3%)	25 (61%)
Fatigue	7 (64%)	0 (0%)	17 (57%)	1 (3%)	24 (58%)
Anorexia	4 (36%)	0 (0%)	16 (53%)	1 (3%)	20 (49%)
Rash	4 (36%)	0 (0%)	16 (53%)	0 (0%)	20 (49%)
Vomiting	4 (36%)	0 (0%)	15 (50%)	0 (0%)	19 (46%)
Pain- abdominal	6 (54%)	0 (0%)	9 (30%)	0 (0%)	15 (37%)
Epistaxis	0 (0%)	0 (0%)	8 (27%)	0 (0%)	8 (19%)
Dysgeusia	3 (28%)	0 (0%)	7 (23%)	0 (0%)	10 (24%)
Palmoplantar Erythrodysesthesia (PPD)	6 (54%)	0 (0%)	7 (23%)	1 (3%)	13 (32%)
Pain- back	3 (27%)	0 (0%)	5 (17%)	2 (7%)	8 (19%)
Peripheral neuropathy	1 (9%)	0 (0%)	5 (17%)	0 (0%)	6 (15%)
Liver transaminase (AST, ALT) elevation	0 (0%)	0 (0%)	4 (13%)	1 (3%)	4 (10%)
Chills	1 (9%)	0 (0%)	4 (13%)	0 (0%)	5 (12%)
Dyspnea	0 (0%)	0 (0%)	4 (13%)	1 (3%)	4 (10%)
Flatulence	3 (28%)	0 (0%)	4 (13%)	0 (0%)	7 (17%)
Hypokalemia	0 (0%)	0 (0%)	4 (13%)	2 (7%)	4 (10%)
Anemia	3 (28%)	1 (9%)	2 (7%)	2 (7%)	5 (12%)

AST: aspartate aminotransferase, ALT: alanine aminotransferase.

Supplemental Table S2. Genomic Profile and Best Response.

**Tumor**	***EGFR/HER2/HER3***	***KRAS***	***TP53***	**Cell Cycle**	**Others**	**ORR**
BTC	*EGFR* amp	*KRAS*^{WT}	*TP53* exons 2-11 loss		*PBRM1* S652<br>*FAM123B* Q457	uPR
BTC		*KRAS*^{WT}	*TP53* F113L	*CDK6* amp	*IDH2* R172K<br>*NRAS* N185S	SD
BTC	*HER2* amp	*KRAS*^{WT}			*CTNNB1* S45P	SD
BTC		*KRAS*^{WT}			*IDH2* R172S<br>*SETBP1* K1546Q<br>*CREBBP* T1931P<br>*DNMT3A* W409<br>*ASXL1* V1540G<br>MSS<br>TMB low	SD
BTC		*KRAS* G12D		*CCND1* amp	*GNAS* R201H<br>*FGFR1* amp	SD
PDA		*KRAS*^{WT}				SD

PDA		*KRAS* G12V	*TP53* P27L fs*17	*CCNE1* amp<br>*CDKN2A* Y44X	*ATM* E2221X<br>*RAB39A-ATM* rearrangement<br>ARID1A loss<br>MSS	SD
PDA		*KRAS* G12D			MSS unknown<br>TMB unknown	SD
PDA		*KRAS* G12R	*TP53* R282_R283		*ARID1B* R1845<br>*PBMR1* S1543fs<br>TMB low	SD
BTC	*HER2* amp	*KRAS*^{WT}	*TP53* R273C	*CCNE1* amp	*FBXW7* R479Q<br>*MYC* amp<br>*CDK12* amp	PD
BTC		KRAS^{WT}	TP53 Q192*		FANCA exon 2-12 loss<br>TERT 124 C>T<br>TET2 S1848	PD
BTC		*KRAS*^{WT}	*TP53* splice site 920-1 G>T	*CDKN2A/B* loss	*FANCG* S237<br>*DNMT3A* E616<br>*PDL1* amp<br>*PDL2* amp<br>MSS<br>TMB 10 m/mb	PD
BTC		*KRAS*^{WT}	*TP53* E339			PD
BTC	*EGFR* amp	*KRAS*^{WT}	*TP53* S215I	*CDKN2A*<br>E10 fs*16	*ARID1A Q562* fs<br>*PRKC1* amp<br>*RBM10* K464<br>*TERC* amp<br>MSS<br>TMB low	PD
BTC		*KRAS*^{WT}			MSS<br>TMB low	PD
BTC		*KRAS* G12V	*TP53* S127P	*CDKN2A*<br>*p16INK4a* R80*<br>*p14ARF* P94L	*RNF43* E278<br>MSS<br>TMB low	PD
BTC		*KRAS* G13D		*CDKN2A/B* loss	*IDH1* R132C<br>NF2 loss exons 5-16<br>*MUTYH* G382D<br>MSS<br>TMB low	PD
BTC	*HER3* G284R	*KRAS* G13D		*CDKN2A-NFX1* fusion	*SMAD4* Y301<br>MSS<br>TMB low	PD
BTC	*HER2* S310F	*KRAS* K117N	*TP53* R248Q	*CDKN2A*<br>*457+1G>A* splice mt	*APC G524*<br>*APC* E1464*<br>*EP300* c.1282+1G>A<br>*TERT c.-124C>T*<br>*BRAF* G466V<br>MSS<br>TMB low	PD
PDA		*KRAS*^{WT}	*TP53* splice site 920-4_920del CTAGC		MSS<br>TMB low	PD
PDA		*KRAS* G12D	*TP53* Q100fs* 23		*ESR1* amp	PD

		KRAS amp			*KDM5A* amp<br>MSS	
PDA		*KRAS* G12V	*TP53*^{MUT}	*CDKN2A loss*<br>*CDKN2B* loss	*RNF43* E187 fs*1<br>MSS	PD
PDA		*KRAS* G12D	*TP53* R306	*CDKN2A* E61*		PD
PDA		*KRAS* G12V	*TP53* Q104_Y107del	*CDKN2A*<br>*p16INK4a*<br>V59fs*46<br>*CDKN2A*<br>*p14ARF* S73fs*73	*MYC* amp<br>*ARID1A* D1912fs*3<br>*NTRK3* R459W<br>MSS<br>TMB low	PD
PDA		*KRAS* G12D	*TP53* splice<br>559+1G>A		*MLL2* K866 fs*64	PD
PDA		*KRAS* G12V	*TP53* Y220C	*CDKN2A*<br>*p16INK4a*<br>Y44fs*1	*MUTYH* G382D	PD
PDA		*KRAS* Q61K	*TP53* C135W	*CCNE1* amp	*FOXA1* amp<br>*KDM6A* amp<br>*FGFR1* amp<br>MSS<br>TMB low	PD
PDA		*KRAS* G12V	*TP53* E256K	*CDKN2A/B* loss	*MDM2* amp<br>*MAP2K4* loss exons 2-11<br>*CREBBP* H8487Y<br>MSS<br>TMB low	PD
PDA		*KRAS* G12D	*TP53* R248Q	*CDKN2A*<br>*p16Ink4a*<br>L16fs*10	MSS<br>TMB low	PD
PDA		*KRAS* G12V	*TP53* R248Q		MSS<br>TMB low	PD
PDA		*KRAS* G12D	*TP53* R273C		IGF1R mut	PD
PDA		*KRAS* G12D	*TP53* R175H	*CDKN2A*<br>*p16INK4a*<br>L130Q	*AURKA* amp<br>GNAS amp<br>ZNF217 amp<br>MSS<br>TMB low	PD
HCC		*KRAS*^{WT}				PD
GCA		*KRAS*^{WT}		*CDK6* amp	SMAD4 V387<br>*STK11* L252R fs,<br>*PIK3CA* D538N<br>*ATM* 5005-1 G>T	PD
ESO	*HER2* T862A	*KRAS*^{WT}	*TP53* P250L		*MAGI2 1104-1* G>C splice	PD
BTC					NE-insufficient tumor	PD
PDA					NE-10% tumor content	NE
PDA		*KRAS* G12V				NE
BTC	*HER2* amp blood	*KRAS*^{WT}			*PBRM1* S275*	NE

		TP53 R273 blood		*LMO1* C115R	
PDA	*KRAS* G12D	*TP53* Y220C	*CCNE1* amp	*AKT2* amp<br>*KEAP1* V214L<br>*SMAD4* R400 fs*5<br>MSS<br>TMB low	NE
BTC	*KRAS*^{WT}	*TP53* R282W	*CCNE1* amp	*AKT2* amp<br>*PIK3CA* E545K<br>*RUNX1* exons 1-6	NE

Abbreviations: amp, amplification; BTC, biliary tract cancer; GCA, gastric adenocarcinoma; ESO, esophageal adenocarcinoma; fs, frameshift; HCC, hepatocellular carcinoma; MSS, microsatellite stable; mut, mutation; NE, not evaluable; PD, progressive disease; PDA, pancreatic ductal adenocarcinoma; SD, stable disease; TMB, tumor mutation burden.
